# Lcz696 Alleviates Myocardial Fibrosis After Myocardial Infarction Through the sFRP-1/Wnt/β-Catenin Signaling Pathway

**DOI:** 10.3389/fphar.2021.724147

**Published:** 2021-09-02

**Authors:** Jing Liu, Xuehui Zheng, Chen Zhang, Chunmei Zhang, Peili Bu

**Affiliations:** ^1^ The Key Laboratory of Cardiovascular Remodeling and Function Research, Chinese Ministry of Education, Chinese National Health Commission and Chinese Academy of Medical Sciences, The State and Shandong Province Joint Key Laboratory of Translational Cardiovascular Medicine, Department of Cardiology, Qilu Hospital, Cheeloo College of Medicine, Shandong University, Jinan, China; ^2^ Department of Cardiology, Heze Municipal Hospital, Heze, China

**Keywords:** myocardial infarction, myocardial fibrosis, LCZ696, Wnt/β-catenin signaling, SFRP-1

## Abstract

**Background:** Lcz696 (ARNI, angiotensin receptor–neprilysin inhibitor; sacubitril/valsartan) shows an inhibitory effect on fibrosis after myocardial infarction (MI). However, the underlying signaling mechanisms are poorly understood. The Wnt/β-catenin signaling pathway is activated after MI and participates in the process of myocardial fibrosis. Here, we aimed to assess the efficacy of ARNI for alleviating myocardial fibrosis after MI and hypothesized that ARNI alleviates myocardial fibrosis by inhibiting the Wnt/β-catenin signaling pathway and overexpressing sFRP-1, an inhibitor of the Wnt/β-catenin signaling pathway.

**Methods:** Mice randomized at 1 week post-MI were administered lcz696 (60 mg/kg, n = 21), valsartan (30 mg/kg, n = 19), or corn oil (n = 13) orally for 4 weeks, while the sham-operated group received vehicle (corn oil, n = 19). Cardiac function and extent of myocardial fibrosis were measured. Western blotting and quantitative real-time polymerase chain reaction were used to detect the expression of Wnt/β-catenin pathway-related proteins. Furthermore, primary myocardial fibroblasts were stimulated with angiotensin II (Ang II) and cultured with lcz696 and the sFRP-1 inhibitor way316606 to detect the expression of Wnt/β-catenin pathway proteins.

**Results:** Both lcz696 and valsartan alleviated myocardial fibrosis and improved cardiac function, but lcz696 had superior efficiency compared to valsartan. Furthermore, β-catenin expression was inhibited and sFRP-1 was overexpressed after drug treatment, which could be significantly improved by lcz696 in mice. In addition, lcz696 inhibited β-catenin expression in AngII-stimulated myocardial fibroblasts, and β-catenin expression increased after the inhibition of sFRP-1.

**Conclusion:** ARNI alleviated cardiac fibrosis and cardiac remodeling by inhibiting the Wnt/β-catenin signaling pathway. In addition, ARNI can lead to overexpression of sFRP-1, which is an inhibitor of the Wnt/β-catenin signaling pathway. These results indicate a new therapeutic target of ARNI to improve myocardial fibrosis and prevent myocardial remodeling.

## Introduction

Heart failure following acute myocardial infarction (MI) remains a major public health concern worldwide, exerting a substantial economic burden (Mozaffarian et al., 2016). Cardiac remodeling is viewed as a key determinant of the clinical outcomes in heart diseases. Cardiac fibrosis, which is typically seen in the failing heart, is a major aspect of the remodeling process. Myocardial fibrosis is an important pathophysiological process observed after MI (Zile et al., 2019). Cardiac fibrosis arises from a pathological attempt to repair tissue damage during maladaptive remodeling. The proliferation of interstitial fibroblasts and increased deposition of extracellular matrix components result in myocardial stiffness and diastolic dysfunction, which ultimately leads to heart failure. Treatment options to block or reverse fibrosis are scarce (Burke et al., 2019). Lcz696 (valsartan/sacubitril), the first of the new ARNI (dual-acting angiotensin-receptor–neprilysin inhibitor) drug class, contains equimolar amounts of valsartan which is an angiotensin-receptor blocker, and sacubitril, which is a prodrug for the neprilysin inhibitor, and ARNI has been extensively reported to approve for the treatment of heart failure patients with reduced ejection fraction [ (Campbell, 2017). (Voors et al., 2013)]. ARNI, apart from blocking angiotensin II (AngII)-signaling, also augments natriuretic peptides by inhibiting their breakdown by neprilysin (von Lueder, 2015), an endopeptidase that degrades various vasoactive peptides, including ANP (atrial natriuretic peptide) and BNP (brain natriuretic peptide) (Burke et al., 2019). Natriuretic peptides, activated in cardiac dysfunction and HF, counteract the RAAS and promote vasodilation, natriuresis, and inhibit fibrosis and hypertrophy. The addition of the neprilysin component in lcz696 augments plasma levels of natriuretic peptides (Voors et al., 2013). In fact, the PARADIGM-HF trial compared enalapril and sacubitril/valsartan (ARNI) therapies and showed that the latter can significantly reduce cardiovascular death and hospitalization in patients with systolic heart failure (McMurray et al., 2014a). ARNI reportedly can improve cardiac function and remodeling; however, the mechanisms underlying the cardioprotective action of ARNI in the context of fibrosis and remodeling after MI are mostly unknown. Despite recent formal recognition of ARNI by guideline authorities, there is a striking paucity of mechanistic data on the effect of ARNI compared to those on stand-alone RAAS blockade on cardiac remodeling (Ponikowski et al., 2016).

Regarding the underlying molecular mechanisms, ample studies have recently shown an emerging role of the Wnt/β-catenin signaling pathway in the process of cardiac remodeling; inhibition of this pathway has been shown to be cardioprotective, to avert cardiac inflammation and fibrosis, to reduce infarct size and scaring, and to stimulate functional recovery of the heart (Eid et al., 2020).

Wnt signaling pathways are considered essential in heart development and are found to be active in the post-MI heart (Voors et al., 2013; McMurray et al., 2014a). Wnt/β-catenin signaling in the adult heart is quiescent under normal conditions; however, it is reactivated after MI, playing a dominant role in the regulation of cardiac fibrosis [ (Palevski et al., 2017). (Fu et al., 2019)]. The expression of several Wnt pathway proteins, such as β-catenin and Dvl-1, is increased in the damaged tissues after experimental MI induction (Barandon et al., 2003a; Chen et al., 2004; Barandon et al., 2005; Kobayashi et al., 2009). In addition, Wnt/β-catenin signaling has been shown to promote cardiac fibrosis by inducing the transition of endothelial and epicardial cells into a mesenchymal state, differentiation of fibroblasts into myofibroblasts, and production of collagen (Mizutani et al., 2016). Importantly, several studies have shown that the inhibition of Wnt/β-catenin signaling after MI is beneficial, as it improves infarct healing and prevents heart failure. This suggests that blocking the Wnt/β-catenin signaling pathway could be a potential novel therapeutic approach to prevent adverse cardiac remodeling after MI.

The secreted frizzled-related protein 1 (sFRP-1), an antagonist of the Wnt/β-catenin pathway (Tao et al., 2015), has been shown to significantly inhibit the proliferation of cardiac fibroblasts, synthesis of collagen, and differentiation of myofibroblasts, and to consequently effectively alleviate the progression of pathological cardiac fibrosis (Sklepkiewicz et al., 2015). Importantly, Barandon et al. demonstrated the upregulation of sFRP-1 in the heart after MI (Ge et al., 2019).

Mechanistically, the beneficial impact of ARNI may partially stem from the inhibition of Wnt/β-catenin signaling. Based on previous studies, we hypothesized that lcz696 could be implicated in post-MI myocardial fibrosis by regulating the Wnt/β-catenin signaling pathway. However, the correlation between ARNI and sFRP-1 has not been studied thus far. Therefore, herein, we evaluated the effects and mechanisms of ARNI in the context of MI, both *in vitro* and *in vivo*. Particularly, we comprehensively investigated the relationship among ARNI, the Wnt//β-catenin pathway, and sFRP-1.

## Materials and Methods

### Animal Protocols

All mouse studies were approved by the Animal Ethics Committee of Shandong University; the care and use of animals followed the guidelines on animal ethics. Eight-week-old C57BL/6J male mice were purchased from SBF Biotechnology Co. Ltd. (Beijing, China). The mice were housed at a constant temperature (24°C) and provided a normal diet with free access to water. C57BL/6J male mice underwent either a permanent coronary artery ligation to induce MI or sham surgery. Briefly, the left coronary artery (LCA) was positioned using a 6–0 silk suture, stitched, and ligated approximately 3 mm from its starting point (Gao et al., 2010). When the front wall of the left ventricle (LV) turned pale, ligation was considered successful. The sham group received the same surgical procedure but without blocking the LCA. One week following LCA ligation, mice were assessed by echocardiography for study inclusion. Mice were randomly assigned into one of four following groups: sham-operated group (Sham, n = 20; 19 alive), MI group (MI, n = 20; 13 alive), VAL group (VAL; n = 40; 21 alive), and ARNI group (ARNI; n = 40; 19 alive). After a week of adaptive feeding, surviving mice were administered VAL or ARNI via gavage. Some studies have proposed excessive toxic side effects of ARNI. Therefore, we referred to the experimental study of Suematsu et al. and adopted the administration of ARNI (60 mg/kg) or VAL (30 mg/kg) dissolved in corn oil or only corn oil every day in the morning (8–9 AM) for 4 weeks. Moreover, the dose of lcz696 (60 mg/kg/day) was selected based on a previous report (Xia et al., 2017). Of note, there was no difference in feeding conditions among all mice.

### Cardiac Function Measurement

Mice were anesthetized via inhalation of isoflurane gas; induction was performed at 2.5% in a chamber, and maintenance was carried out at 1.5% isoflurane via a nose adaptor. The cardiac function was then evaluated using the Vevo770 imaging system (Visual Sonic, Toronto, Canada). The left ventricular ejection fraction (LVEF), fractional shortening (FS), early (E) peak, late (A) peak, left ventricular end-diastolic dimension (LVEDd), and left ventricular mass (LV mass) were measured. The ratios of early-to-late mitral flow velocity (E/A) and diastolic velocity ratio (E’/A’) were also calculated. Additionally, the internal diameter of the LV and the thickness of the septum and posterior wall at end-systole and end-diastole were measured from the long-axis view at the level of chordae tendineae. LVEF and FS were then calculated according to these parameters. Echocardiographic imaging and measurements were performed in a blinded manner. All measurements were averaged based on three consecutive cardiac cycles.

### Serum NT-proBNP and Blood Pressure Measurements

The serum levels of NT-proBNP were measured using a commercial enzyme-linked immunosorbent assay (ELISA)—Mouse NT-proBNP ELISA Kit (MyBioSource, CA, United States), according to the manufacturer’s instructions. Briefly, this assay employs a quantitative sandwich enzyme technique. A microplate is pre-coated with an antibody specific for NT-proBNP; the analyte is captured, and then sandwiched with a biotin-conjugated antibody. Thereafter, avidin-conjugated horseradish peroxidase is added, followed by a specific substrate. Finally, color development is stopped, and the intensity of the color is measured. In our assay system, the intensity of the developed color was positively correlated with the concentration of mouse NT-proBNP in the sample, and the absorbance (OD value) was measured with using an absorbance microplate reader (SpectraMax plus 384, American Molecular Devices Scientific Company) at 450 nm wave length.

The systolic and diastolic blood pressure of awake mice was measured using the tail-sleeve method before and after surgery and before death. The heart rate (HR), systolic blood pressure (SBP), and diastolic blood pressure (DBP) were measured using a noninvasive tail-cuff system (Softron BP-98A, Softron, Tokyo, Japan).

### TTC Staining

Animals were humanely euthanized, and their hearts were quickly removed, rinsed with normal saline, and dried. Heart tissues were frozen in a −20°C refrigerator for 30 min until they hardened. Then, from the apex of the heart to the bottom, 1–2 mm thick sections were obtained, along the direction of the atrioventricular groove. A total of five slices were cut. The slices were quickly placed into a TTC phosphoric acid buffer solution (Solarbio, Beijing, China) at 37°C, and were subsequently bathed in water for 15 min. The infarcted area after staining appeared white, while the normal areas appeared red.

### Histology and Immunohistochemistry

Mice were anesthetized and euthanized, and their hearts were dissected; the dissected tissues were immediately fixed with 4% formalin. Tissues were then embedded within paraffin and sectioned (5 µm) for histology staining including hematoxylin and eosin (H&E) and immunohistochemistry. Of note, the cardiac tissues were removed from 3 to 4 mm below the ligation site to ensure that the pathological sections of cardiac tissues were at the same level. For the detection of interstitial collagen deposition, heart sections were stained with PicroSirius Red and Masson’s trichrome according to the manufacturer’s instructions. PicroSirius Red staining images were captured at 10× on a BX51 epifluorescence microscope (Olympus, Shinjuku, Japan) using circularly polarized light to generate a birefringent (red/thick and green/thin) signal from collagen fibers. To evaluate fibrosis, the areas of interest (epicardial, perivascular, and interstitial) were defined using ImageJ. The fraction of the fibrosis area was quantified using ImageProPlus6.0 (Media Cybernetics, Sarasota, FL, United States).

For immunohistochemistry, the slides were incubated overnight at 4°C with specific primary antibodies against SFRP-1 (1:800, Abcam, Cambridge, United Kingdom), β-catenin (1:50, Cell Signaling Technology, Leiden, Germany), GSK-3β (1:50, Santa Cruz Biotechnology, TX, United States), collagen I (1:200, Abcam), and collagen III (1:500, Abcam). The next day, the samples were incubated with secondary antibodies and diaminobenzidine staining (ZSGB-Bio, Beijing, China) was performed following the manufacturer’s protocols. Data were analyzed using ImageProPlus6.0 (Media Cybernetics, Sarasota, FL, United States).

### TUNEL Assay

Apoptotic cells in myocardium were detected using a *in situ* cell death detection kit (Roche, 12156792910) following the manufacturer’s instructions. Heart sections were deparaffinized, hydrated, kept in permeabilization solution (0.1% Triton X-100) for 5 min, and incubated in TUNEL reaction mixture for 60 min at 37°C; the sections were then sealed in Prolong Gold Anti-Fade Reagent with 4-6-diamidino-2-phenylindole (DAPI, Invitrogen). Images were acquired via a fluorescence microscopy (Nikon) and quantified using ImageProPlus6.0 software (Media Cybernetics, Sarasota, FL, United States).

### Immunofluorescence

Heart sections were incubated with specific primary antibodies against α-SMA (Abcam, Cambridge, MA, United States) at the appropriate concentrations at 4°C overnight. The next day, horseradish peroxidase-conjugated secondary antibodies were added for 1 h at 37°C. Nuclei were visualized using DAPI staining. Fluorescent images were acquired via a fluorescence microscopy (Nikon) and quantified using Image Pro Plus 6.0 software (Media Cybernetics, Sarasota, FL, United States).

### Cellular Cardiac Fibrosis *in vitro* Model

Primary myocardial fibroblasts were obtained from 2–3-day-old mice via enzyme digestion and differential plating (Burke et al., 2019). Briefly, ventricles were isolated and transferred into a solution containing 0.8 mg/ml collagenase type II (Solarbio) and finely minced. Tissues were then agitated in 4–6 ml of collagenase solution in four cycles of 60 min each, and cells were collected at each step by centrifugation. Cells were then strained through 70-μm filters and plated onto 6-well plate for 2 h. Non-adherent cells were then removed, and the adherent cells were considered fibroblasts. The culture medium was then replaced, and the cells were maintained in DMEM (Solarbio) containing 10% FBS and penicillin/streptomycin (Invitrogen, Carlsbad, CA, United States) in a 5% CO_2_ humidified incubator at 37°C for 36 h. Before lcz696 or VAL stimulation, the cells were starved in serum-reducing medium (2% FBS) for 12 h. Subsequently, we added different lcz696 (ARNI, HY-18204A; MCE) in varied concentrations (1, 10, and 30 μmol/L) (Ge et al., 2019) and incubated the cells for 1 h; Val (HY-18204; MCE) (30 μmol/L) was used as the positive control. After repeated experiments, the optimal concentration of lcz696 (30 μmol/L) was finally determined. Additionally, to investigate the potential role of sFRP-1, we incubated cells overnight with the sFRP-1 inhibitor way316606 (2 μmol/L; MCE, HY-10858). The stimulation of collagen synthesis with Angiotensin II (*AngII, Sigma*) 100 nmol/L requires 48 h (von Lueder, 2015). Therefore, cells were treated as mentioned above, and only then received AngII, followed by 2 days of incubation. Experiments were repeated at least three times.

### Quantitative Real-Time Polymerase Chain Reaction (qRT-PCR) Assay

Total RNA was extracted from the mouse hearts using the TRIzol reagent (Ambion) following the manufacturer’s protocol, and the conversion of mRNA into cDNA was performed using the Primescript™ RT reagent kit with gDNA Eraser (RR047A, Takara, Tokyo, Japan). qRT-PCR was performed using SYBR Premix Ex TaqII (RR420A; Takara) in the iQ5 Multicolor Real-Time PCR Detection System (Bio-Rad, Hercules, CA, United States) using gene specific primers (Table 1). Relative mRNA expression was quantitated by 2^−ΔΔCt^ comparative quantification method (Liu et al., 2019). All experiments were repeated at least three times.

**TABLE 1:** Information of primers for RT‐qPCR.

Gene name	Forward sequence (5′–3′)	Reverse sequence (5′–3′)
β-catenin	5’TTG​TAG​AAG​CTG​GTG​GGA​TGC	5’AGT​CGC​TGC​ATC​TGA​AAG​GT
sFRP-1	5’GCG​AGT​TTG​CAC​TGA​GGA​TG	5’GTT​GTG​GCT​GAG​GTT​GTC​CA
α-SMA	5’TTC​GTG​ACT​ACT​GCC​GAG​C	5’GTC​AGG​CAG​TTC​GTA​GCT​CT
GSK-3β	5’GAG​CCA​CTG​ATT​ACA​CGT​CCA	5’CCA​ACT​GAT​CCA​CAC​CAC​TGT
TGF-β	5’AGC​TGC​GCT​TGC​AGA​GAT​TA	5’AGC​CCT​GTA​TTC​CGT​CTC​CT
ANP	5’CTC​CGA​TAG​ATC​TGC​CCT​CTT​GAA	5’GGT​ACC​GGA​AGC​TGT​TGC​AGC​CTA
BNP	5’GCT​CTT​GAA​GGA​CCA​AGG​CCT​CAC	5’GAT​CCG​ATC​CGG​TCT​ATC​TTG​TGC

### Western Blot Analysis

Total protein from mouse myocardium tissues and primary cardiac fibroblasts was extracted using a protein extraction kit and protein concentration was measured using BCA Protein Assay kit (Beyotime, China) according to the manufacturer’s protocol. The proteins were separated by 10% gradient SDS-polyacrylamide gel electrophoresis (SDS-PAGE) and transferred to a polyvinylidene difluoride (PVDF) membrane using the wet transfer method. The membranes were blocked with 5% defatted milk for 1 h at room temperature and then incubated overnight at 4°C with specific primary antibodies against sFRP-1 (1:230,Abcam, ab4193), β-catenin (1:1,000, Cell Signaling Technology, D10A8), active β-catenin (1:1,000, EMD Millipore, 05–665), GSK-3β (1:1,000, Santa Cruz Biotechnology, 0011-A),p-GSK-3β (1:1,000, Cell Signaling Technology, 5558T), α-tubulin (1:10,000,Abcam, ab7291), collagen III (1:5,000,Abcam,ab7778) and α-SMA (1:10,000, Cell Signaling Technology, 19245s). All primary antibodies are diluted in proportion with antibody diluent (Boster). The membranes were washed and incubated with the anti-mouse/rabbit secondary antibodies labeled with horseradish peroxidase (1:5,000; ZSGB-Bio, Beijing, China) for 1 h at room temperature. The blots were subsequently detected using a chemiluminescence kit (Millipore, Billerica, MA, United States) as per the manufacturer’s instructions. All experiments were repeated at least three times.

### Statistical Analysis

All data are expressed as mean ± SEM derived from at least three independent experiments. Statistical analysis was performed using GraphPad Prism 8 (GraphPad, San Diego, CA, United States). The Shapiro-Wilk test was applied for normality assumption. Thereafter, normally distributed data were analyzed using the unpaired two-tailed Student’s *t*-tests (two-group comparisons), or the one-way ANOVA followed by Dunnett’s or Tukey’s post hoc tests (comparisons between multiple groups). *p* < 0.05 was considered statistically significant.

## Results

### Anti-myocardial Remodeling Effect of ARNI Is Superior to That of Valsartan

**FIGURE 1:**
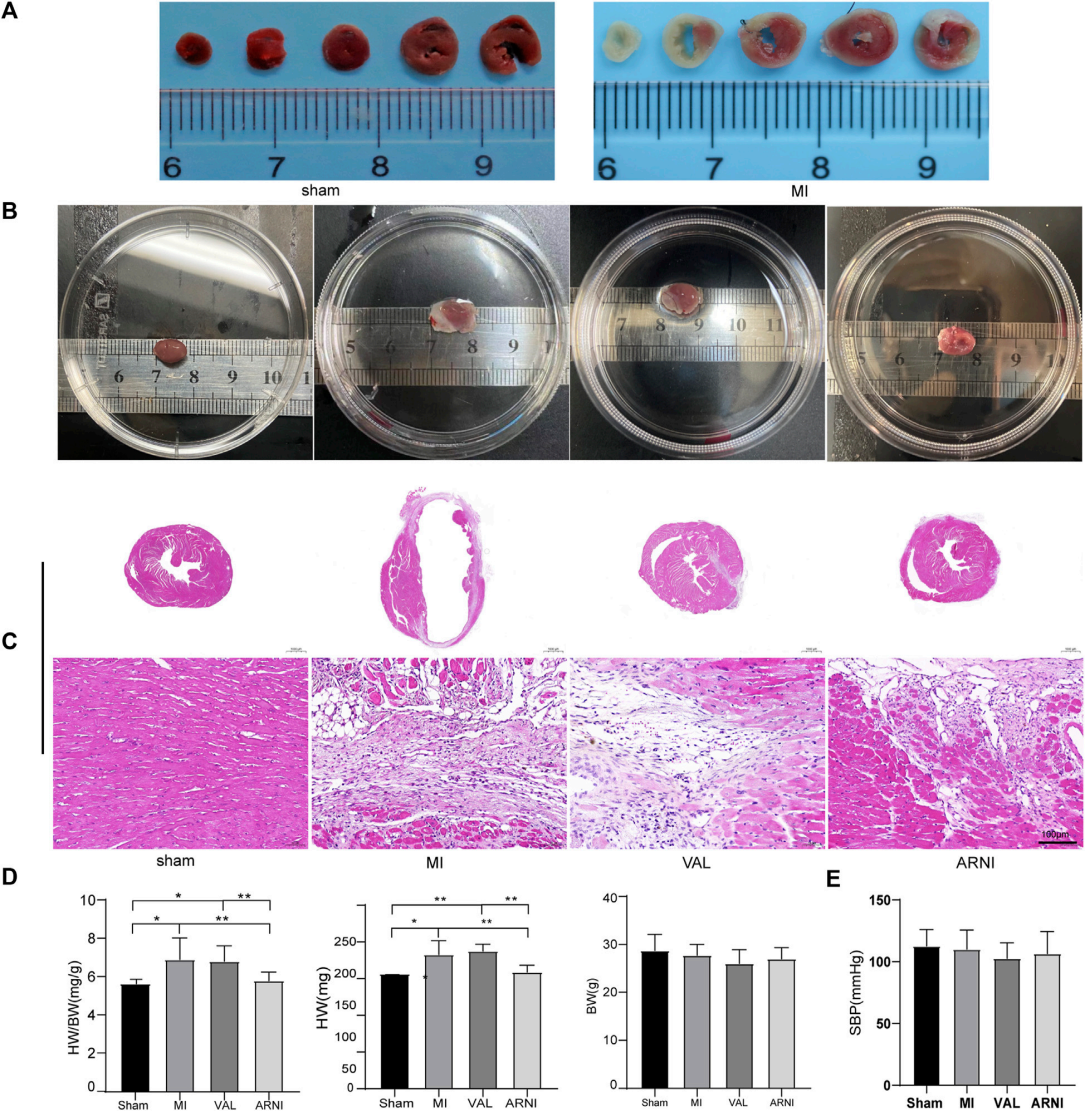
TTC staining, heart size, and pathology in the four groups of mice. **(A)** TTC staining.**(B)** The heart gross morphological image. **(C)** H&E staining of Cross-sections of the hearts at the papillary muscle level and the infarct section (scale bar: 100 μm). **(D)** Quantitative analysis of the heart weight to body weight (HW/BW) ratio. **(E)** SBP-systolic blood pressure. Sham: sham-operated group; MI: myocardial infarction; VAL: valsartan intervention group; ARNI: Sacubitril/valsartan intervention group. Each group n = 7. *p < 0.01,**p < 0.05.

C57BL/6J male mice underwent permanent LCA ligation to induce MI. Importantly, TTC staining was performed 24–48 h after MI and showed that the mouse model of MI was successfully established with the infarcted areas appearing in white; of note, no infarcted areas were detected in sham-operated animals (Figure 1A). Additionally, the MI group showed a significantly increased heart size; however, interestingly, after the drug intervention, the heart size was observed to have decreased, especially in the ARNI group (Figure 1B). Furthermore, HE staining showed that ARNI improved the increase in the ventricular cross-sectional area after MI; at high magnification, we observed the texture of the myocardium in the infarcted area to be white gelatinous, while the texture becomes hard, like ground glass. Of note, the improvement after ARNI treatment was considerably greater than that observed after VAL treatment (Figure 1C). As expected, the ratio of heart weight to body weight in the MI group was higher than that in the sham group (Figure 1D); This ratio in the ARNI group was significantly lower than that in the VAL group. Moreover, there was no significant difference in the body weight among the four groups (Figure 1D). Finally, yet importantly, after the drug intervention, there was a downward trend in the systolic blood pressure (SBP) of mice, more obviously in VAL-treated animals, but without statistical relevance (Figure 1E).

### ARNI Alleviates MI-Induced Cardiac Dysfunction

**FIGURE 2:**
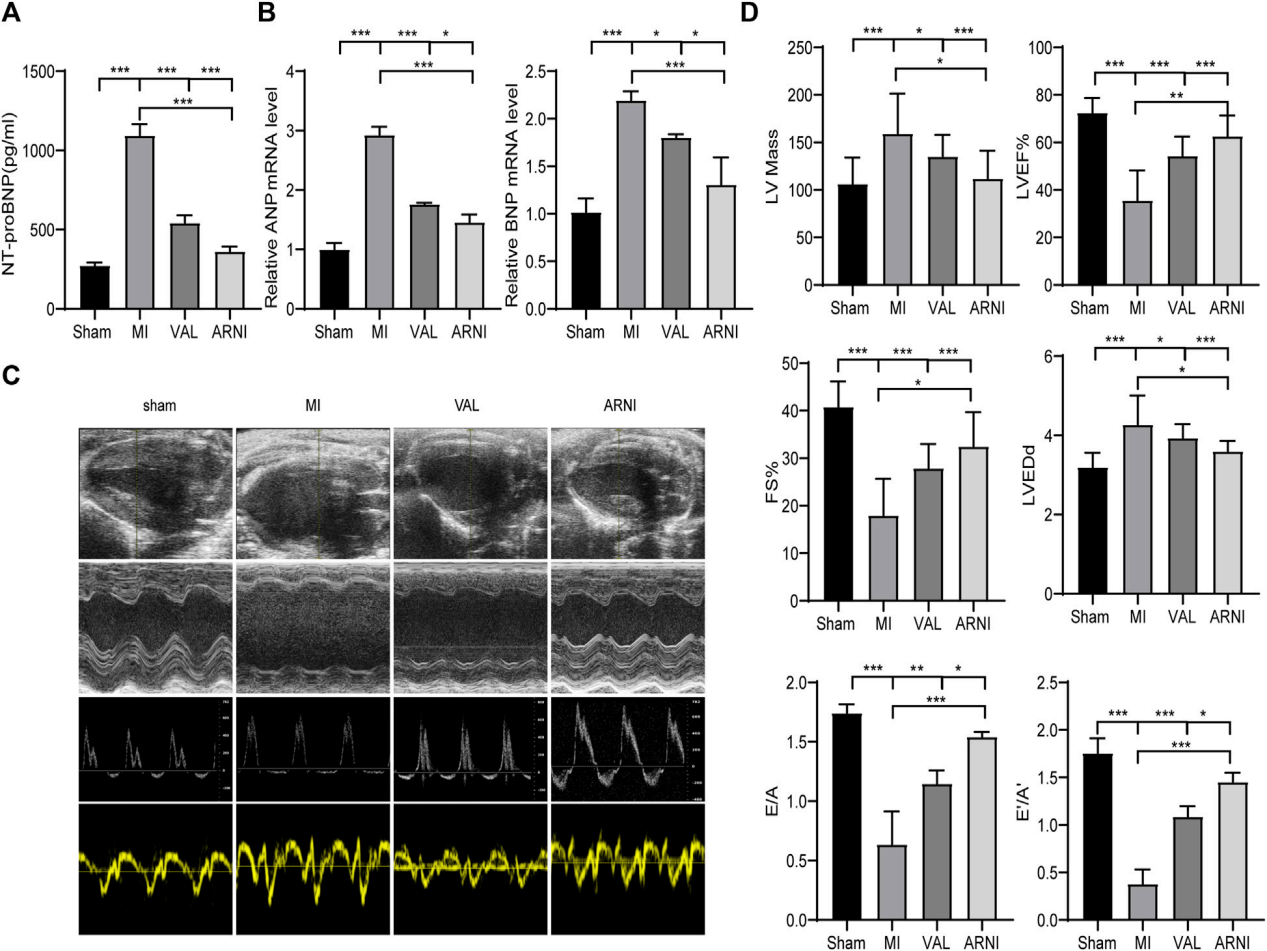
ARNI alleviates cardiac dysfunction in mice with myocardial infarction. **(A)** Serum NT-proBNP concentration. **(B)** Relative mRNA fold changes of *ANP* and *BNP*.**(C)**. The 1st row:Representative 2D echocardiograms. The 2nd row: Representative M-mode echocardiograms. The 3rd row: Representative pulse-wave doppler echocardiograms of mitral inflow. The 4th row: Representative tissue doppler echocardiograms. **(D)** Left ventricular mass, LV ejection fraction (LVEF%), fractional shortening (FS), left ventricular end-diastolic dimension (LVEDd), early-to-late mitral flow (E/A), and diastolic velocity ratio (E’/A’). Each group n = 8.*p < 0.01,**p < 0.05, ***p < 0.001.

The serum NT-proBNP levels were significantly increased 5 weeks after MI in the MI group; of note, there was a significant difference upon treatment with ARNI and VAL, and the effect of ARNI was superior to that of VAL (Figure 2A). Meanwhile, qRT-PCR analysis revealed that the mRNA levels of ANP and BNP in the MI group were significantly increased, while ARNI treatment reversed this trend (Figure 2B). Echocardiography further showed that LVEF, FS, E/A, and E’/A’ in the MI group were decreased compared to those in the sham group and that ARNI treatment significantly improved these metrics; of note, the difference was statistically significant compared with the metrics in the VAL group. Additionally, the LVEDd and LV mass were higher in the MI group than in the sham groups. However, compared with those in the VAL group, the LVEDd and LV mass in the ARNI group showed improvements (Figure 2D).

### ARNI has Anti-fibrosis and Anti-Apoptotic Effects

**FIGURE 3:**
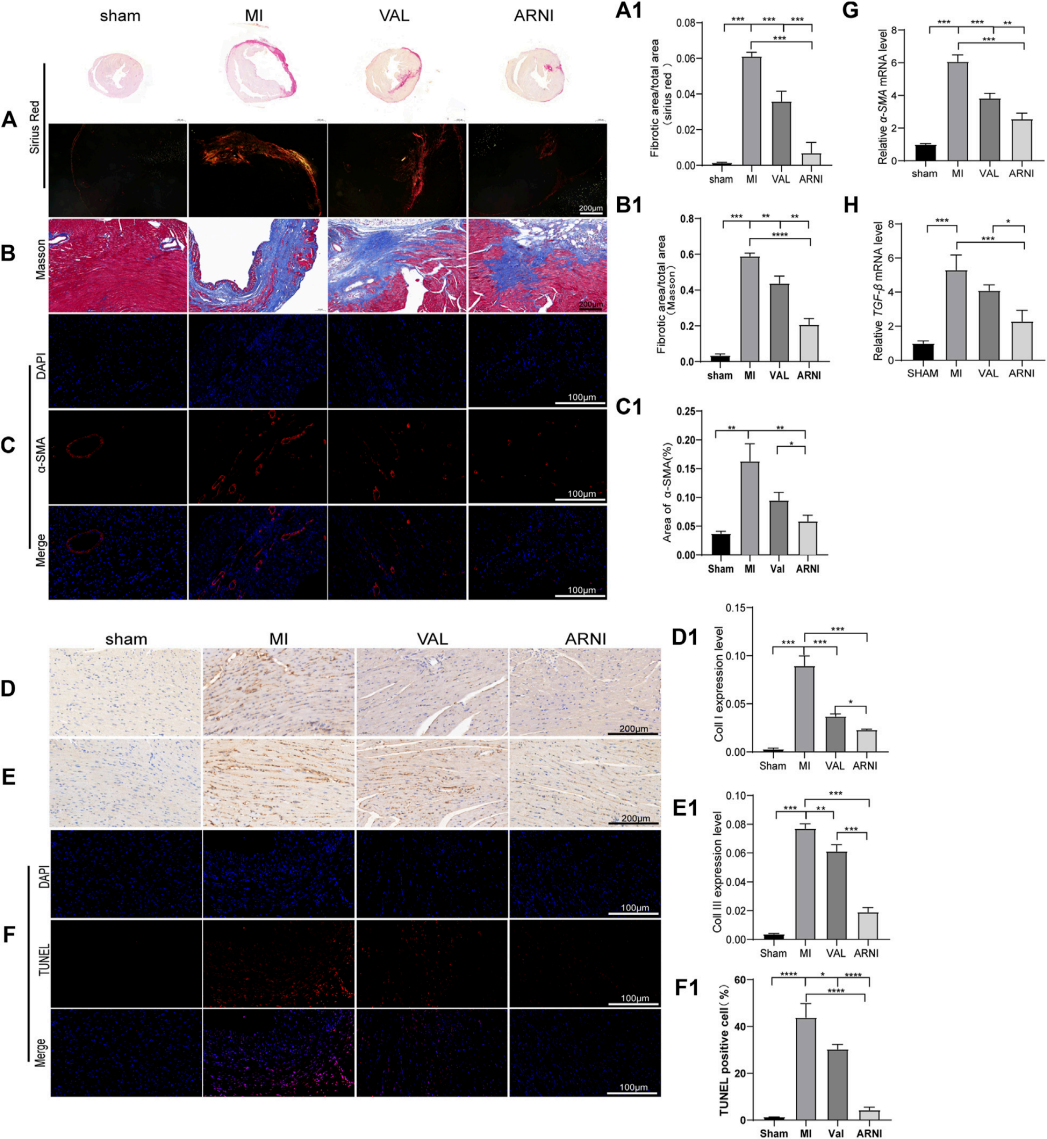
Effects of ARNI on myocardial fibrosis in mice with MI. **(A)** Sirius Red staining of myocardium tissues (2nd row, scale bar = 100 μm). **(A1)** Quantitative analysis of myocardial fibrosis (Sirius Red staining). **(B)** Masson’s trichrome staining of myocardium tissues (scale bar = 200 μm). **(B1)** Quantitative analysis of myocardial fibrosis (Masson’s trichrome staining).**(C)** Immunofluorescence images of myofibroblasts derived from CFs. Red, α-SMA; Blue, nuclei. (scale bars = 100 μm). **(C1)** Quantitative analysis of α-SMA; **(D,D1**,**E,E1)** Immunostaining of collagen I and collagen III. **(F)** TUNEL staining of myocardium (scale bar = 100 μm). **(F1)**. Quantitative analysis of TUNEL positive cells. **(G,H)** Relative mRNA level of α-SMA and TGF-β.Each group n = 7. *p < 0.01,**p < 0.05, ***p < 0.001.

Masson’s trichrome and PicroSirius red staining of heart sections demonstrated that collagen deposition in the hearts of MI animals was worse than that observed in the sham group, as expected. Additionally, ARNI treatment prevented collagen deposition to a greater extent than VAL treatment (Figures 3A,A1,B,B1). We also examined cardiac expression of α-smooth muscle actin (α-SMA), a molecular signature for myofibroblast activation. The immunofluorescence results showed that α-SMA was strongly expressed in the infarct area, and the expression was significantly decreased after drug intervention as shown in Figures 3C,C1. The immunohistochemistry (IHC) results showed that the expression of collagen I and collagen III in and around the infarcted area was enhanced in the MI group and decreased in the ARNI group to a significantly greater extent than that observed in the VAL group (Figures 3D,D1,E,E1). The TUNEL assay showed that the apoptosis rate was also increased significantly in MI group (Figures 3F,F1); apoptosis was found to have decreased after drug intervention, especially for the ARNI group. Meanwhile, qRT-PCR indicated that the mRNA levels of genes encoding for α-SMA and TGF-β in MI animals were significantly increased, and that ARNI treatment reversed this trend; the effects were significantly superior in ARNI-treated animals than in VAL-treated animals (Figures 3G,H). Of note, no significant difference was detected in the expression of TGF-β between the VAL and MI groups.

### Wnt/β-catenin Signaling Pathway Is Activated in Mice From the MI Group

**FIGURE 4:**
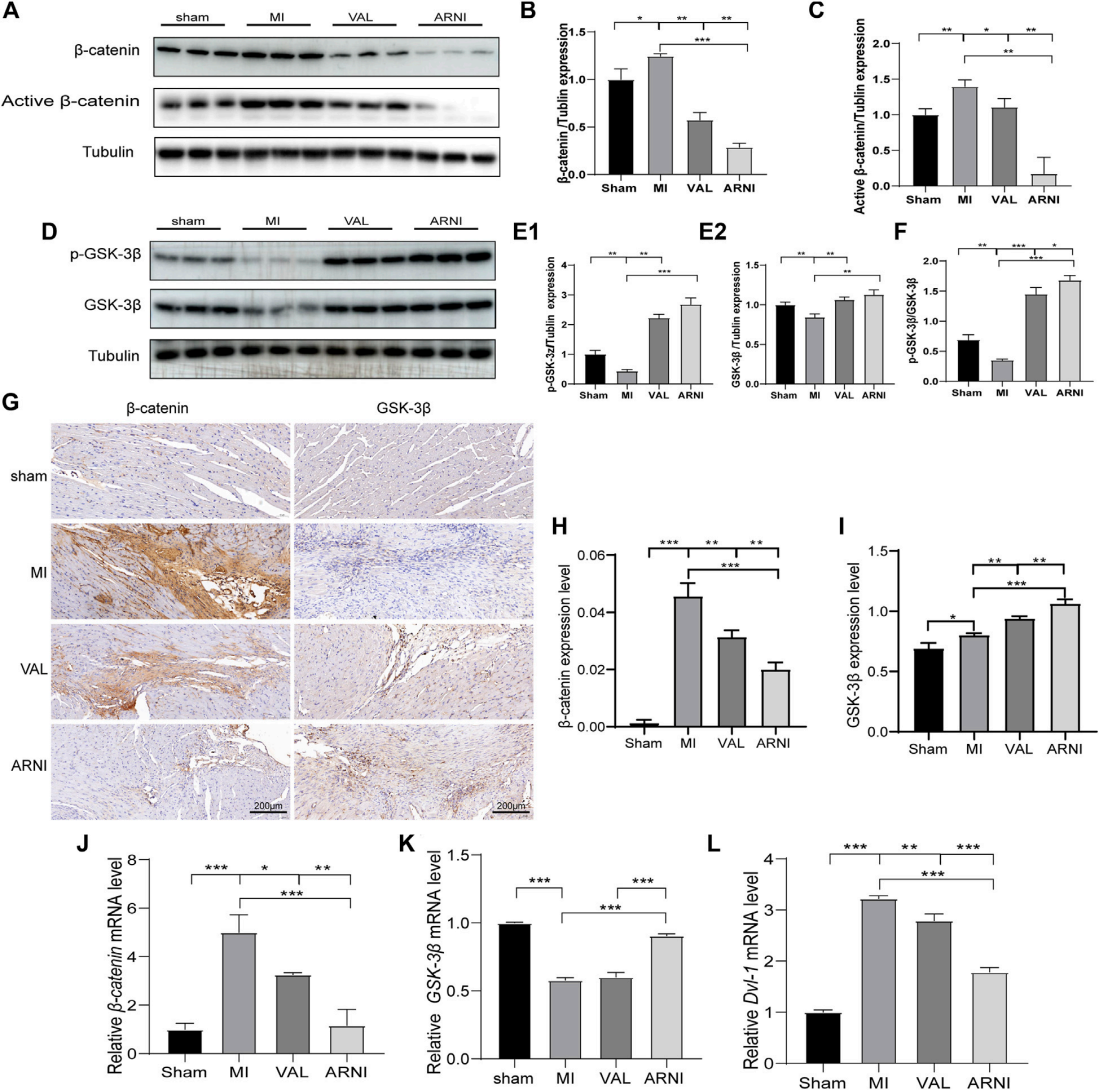
The influence of the Wnt/β-catenin signaling pathway in mice with MI. **(A,B,C).** Western blot analysis of the β-catenin and active β-catenin expression. **(D,E1,E2,F)**. Western blot analysis of the GSK-3β and *p*-GSK-3β expression. G. Immunohistochemistry staining of β-catenin and GSK-3β. **(H, I)** β-catenin and *GSK-3β* Immunohistochemistry quantitative data. **(J,K,L)** Relative mRNA expression level of *β-catenin*,*GSK-3β and Dvl-1*. Each group n = 7. *p < 0.01,**p < 0.05, ***p < 0.001.

Remarkably, western blot analysis showed that the expression of β-catenin and active β-catenin was increased 4 weeks after MI in mice (compared to the sham group). Interestingly, after 4 weeks of treatment with ARNI and VAL, the expression of β-catenin was inhibited; of note, the expression of β-catenin and active β-catenin in the ARNI group was significantly lower than that in the VAL and MI groups (Figures 4A–C). In contrast, the effects on GSK-3β and *p*-GSK-3β were the opposite (Figures 4D–F). Similarly, the IHC results for β-catenin and GSK-3β were also consistent with the above results (Figures 4G–I). Although qRT-PCR results for β-catenin and GSK-3β were also consistent with those stated above (Figures 4J,K), there was no significant difference in the expression of GSK-3β between the VAL and MI groups. Additionally, the expression of Dvl1 was also found to have increased after MI, and decreased after drug treatment, with ARNI outperforming VAL (Figure.4L).

### ARNI Promotes the Expression of sFRP-1

**FIGURE 5:**
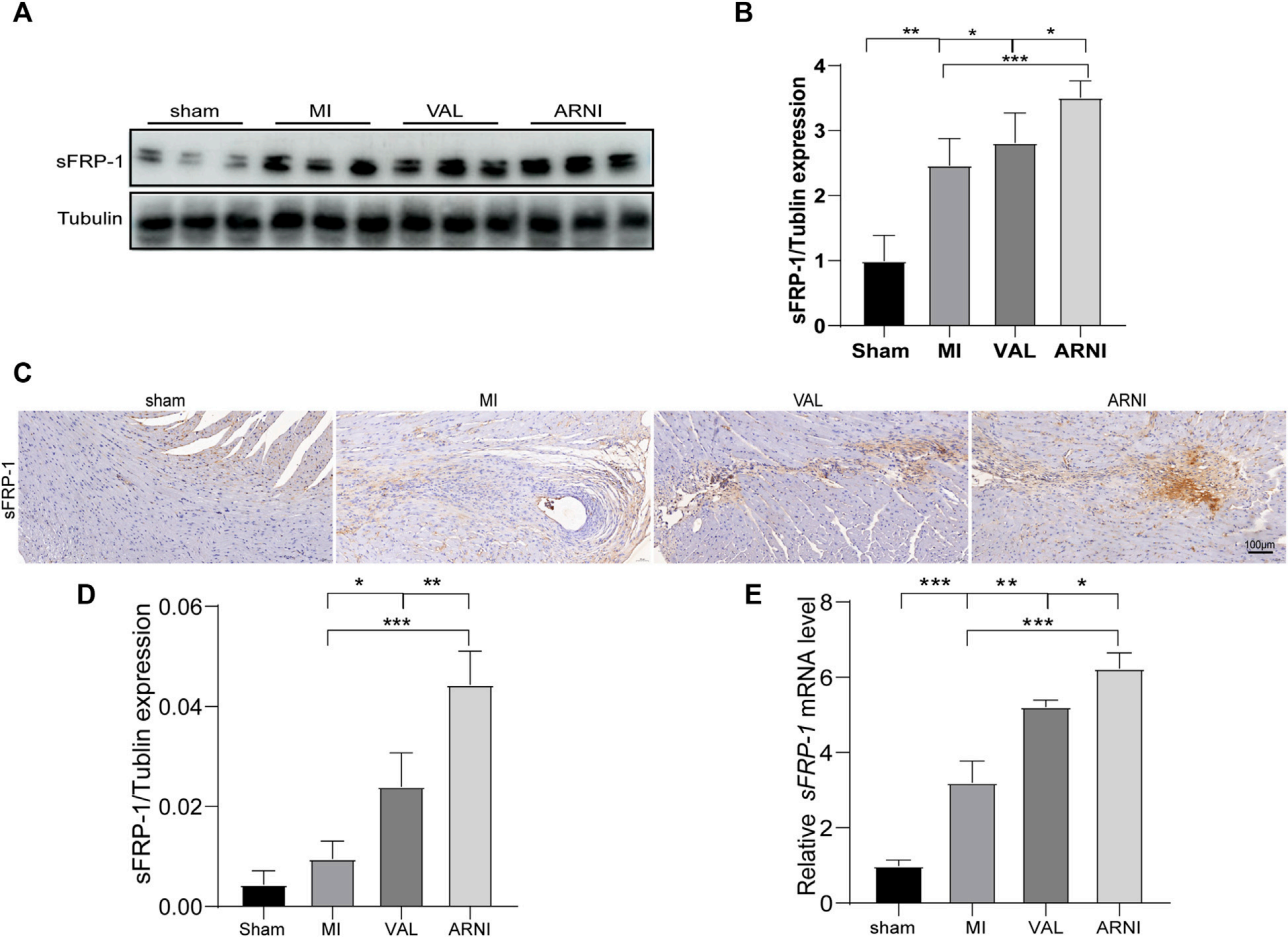
The expression of sFRP-1 after ARNI and VAL intervention. **(A,B)** Western blot analysis of the expression of sFRP-1. **(C,D)** Immunohistochemistry staining of sFRP-1. **(E)** Relative mRNA expression level of sFRP-1. Each group n = 7.*p < 0.01,**p < 0.05, ***p < 0.001.

Next, we investigated the expression of sFRP-1, which is found upstream of the Wnt signaling pathway. The western blot results showed that the expression of sFRP-1 was increased in the MI group compared with that in the sham group. Remarkably, after drug treatment, the expression of sFRP-1 was increased more obvious; of note, the increase was observed to be higher in the ARNI group than in the VAL group (Figures 5A,B). Importantly, the IHC and qRT-PCR analyses yielded similar results; the expression of sFRP-1 was found to have significantly increased in the ARNI group (Figures 5C–E).

### sFRP-1 Regulates the Wnt/β-Catenin Signaling Pathway *in vitro*

**FIGURE 6:**
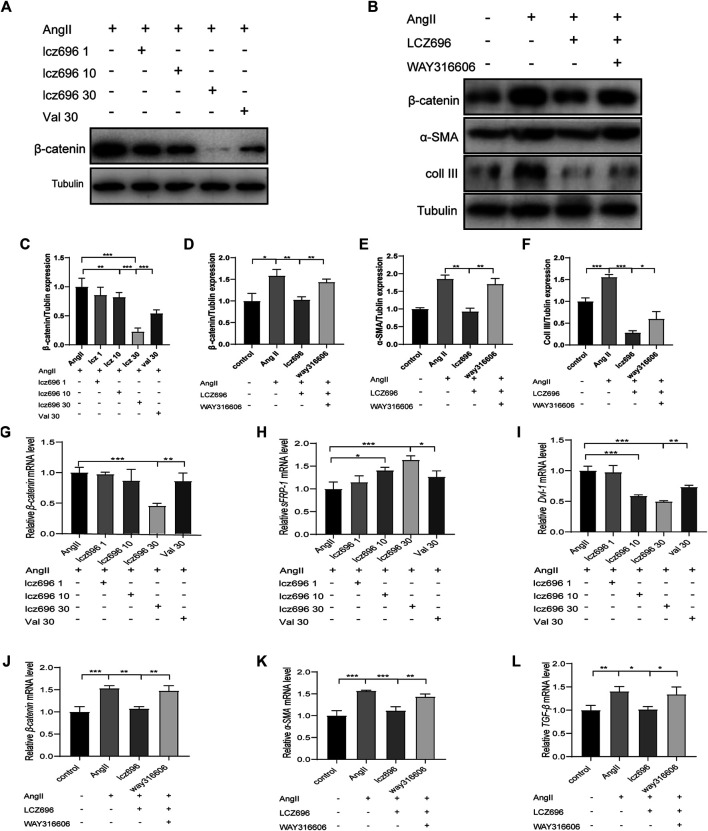
Effects of sFRP-1 on myocardial fibrosis**. (A,C)**Western blot analysis of the β-catenin expression. **(B,D,E,F)**. Western blot analysis of the β-catenin, α-SMA and collagen III expression in the context of way316606 treatment. **(G,H,I).** Relative mRNA expression level of *β-catenin,sFRP-1* and *Dvl-1*.**(J,K,L)** Relative mRNA expression level of *β-catenin, α-SMA* and *TGF-β* in the context of way316606 treatment. Angiotensin II:100 nmol/L; lcz696 1 μmol/L; lcz696 10 μmol/L; lcz696 30 μmol/L; valsartan 30 μmol/L; way316606 2 μmol/L. The experiment was conducted at least 3 times independently,*p < 0.01,**p < 0.05, ***p < 0.001.

For further mechanistic insights, we obtained primary mouse fibroblasts according to the methods mentioned above and studied the effects of lcz696 and VAL on the Wnt/β-catenin signaling pathway as well as on the expression of sFRP-1. The western blot results indicated that β-catenin expression was significantly inhibited at 30 mmol/L lcz696 (Figure 6A). The qRT-PCR results showed that β-catenin expression was significantly inhibited with 30 µmol/L lcz696, while the expression of sFRP-1 was increased (Figures 6G,H). Of note, the qRT-PCR analysis showed that the expression of Dvl-1 was also the lowest at 30 µmol/L lcz696 (Figure 6I). Next, in order to further explore the role of sFRP-1, we used the sFRP-1 inhibitor way316606. Interestingly, western blot analysis revealed that the expression of β-catenin in way316606-treated cells (way316606^+^) was higher than that in lcz696-treated cells and lower than that in AngII-treated cells, suggesting that the influence of lcz696 on β-catenin is related to the expression of sFRP-1 (Figure 6B). The results of qRT-PCR were once again consistent with these observations (Figure 6J). Additionally, qRT-PCR revealed that the expression of α-SMA and TGF-β was significantly increased in AngII-stimulated cells and decreased in lcz696-treated cells; furthermore, their expression in way316606-treated cells was higher than that in lcz696-treated cells (Figure 6K,L). In addition, western blot analysis revealed that the expression of collagen III in way316606-treated cells was higher than that in lcz696-treated cells and lower than that in AngII-treated cells (Figure 6B), suggesting that the sFRP-1 can alleviate collagen deposition in myocardial fibroblasts.

## Discussion

In this study, we show that the sFRP-1/Wnt/β-catenin axis plays a role in the prevention of myocardial fibrosis and improvement of myocardial remodeling in the context of ARNI treatment.

Studies have shown that ARNI has an inhibitory effect on fibrosis after MI, but the exact underlying mechanism is not entirely clear. Cardiac fibrosis is a common process in remodeling hearts after MI and is mediated by myofibroblast invasion and collagen deposition (van den Borne et al., 2010). In the present study, we successfully established a MI model in mice as revealed by TTC staining. Importantly, Masson’s trichrome and PicroSirius Red staining showed that the degree of myocardial fibrosis was the highest in the MI group and was decreased in the ARNI- and VAL-treated groups. Von Lueder et al. found that ARNI could improve the LVEDd and LVEF after MI, reduce cardiac weight, and reduce myocardial fibrosis (von Lueder et al., 2015). We hypothesize that ARNI inhibits cardiac fibrosis and consequently prevents the development of cardiac hypertrophy.

ARNI was observed to show significant improvement over standard of care therapy in patients with class II, III, or IV heart failure and reduce the ejection fraction in the PARADIGM-HF trial (McMurray et al., 2014b). In the present study, we compared the changes in cardiac function after MI in four groups of mice and found that cardiac function was significantly reduced in the MI group and improved in the ARNI- and VAL-treated groups; the improvement in the ARNI group was more significant than that observed in the VAL group. We believe that the use of ARNI is advantageous over the use of a single RAAS blocker, which is supported by echocardiographic data, including the ejection fraction, LVIDd, and LV mass.

The Wnt signal transduction pathway is involved in myocardial repair after MI (Blankesteijn et al., 2000). (Schumann et al., 2000). Studies by Yue Z et al. showed that the connection between RAAS and Wnt/β-catenin might not be a one-way path; rather, it appears to be bidirectional and reciprocal. Therefore, RAAS activation and Wnt/β-catenin could form a vicious cycle, leading to myocardial fibrosis; meanwhile, activation of β-catenin can stimulate the expression of ANP and BNP, which are markers for left ventricular dysfunction and cardiac injury (Zhao et al., 2018). We also assessed cardiac expression of ANP and BNP and found that ARNI can reduce the expression of ANP and BNP. Further, studies have shown that Wnt/β-catenin signaling in resident cardiac fibroblasts is required for excessive extracellular matrix gene expression and collagen deposition after cardiac remodeling (Xiang et al., 2017). The results of PicroSirius Red and Masson’s trichrome, α-SMA immunofluorescence, and collagen I, III immunohistochemistry are consistent with this observation.

Under general physiological conditions, the Wnt/β-catenin signaling pathway is inactive, and β-catenin is phosphorylated by the GSK-3β degradation complex following phosphorylation, β-catenin is identified and degraded by the ubiquitin-mediated proteasomal degradation pathway, maintaining low levels of expression in the cytoplasm. The canonical Wnt signaling pathway is activated after MI (MacDonald et al., 2009), proteins from the Wnt family can bind to frizzled receptors. This causes an activation of the signal transduction molecule disheveled (Dvl) which, in turn, inhibits the (GSK-3β), the expression level of β-catenin increased. However, we found that the expression of *p*-GSK-3β was different from the results of previous studies (Gupte et al., 2018), which may be related to the time stage after myocardial infarction (Lal et al., 2014) or likely related different MI areas, such as MI and non-MI areas or maybe lcz696 likely regulating β-catenin and GSK-3β through two independent pathways, this will encourage us to explore further.

this enzyme is responsible for the phosphorylation of β-catenin (van Gijn, 2002). Inhibition of the Wnt signaling pathway can effectively reduce myocardial remodeling and improve cardiac function. In line with these studies, here, we found that the Wnt signaling pathway was activated in the context of MI and that the expression of β-catenin and Dvl-1 was increased. Of note, ARNI treatment prevented the activation of Wnt signaling.

Although studies have shown that inhibiting the Wnt pathway leads to cardioprotective effects in the context of MI in mice, blocking the Wnt signaling pathway has been shown to lead to an increased heart rupture rate (Barandon et al., 2003b). Barandon et al. established an acute MI model using transgenic mice overexpressing sFRP-1 and reported the inhibition of the Wnt pathway after acute MI in mice as well as the consequent reduction in the MI area and rate of heart rupture; thus, sFRP-1 could control the healing process after MI (Alfaro et al., 2010). Therefore, the timely adoption of certain methods to overexpress sFRP-1 after MI can effectively improve the prognosis of MI. Of note, these protective effects are likely due to the inhibition of the over-activation of the Wnt/β-catenin pathway (Alfaro et al., 2010). (Barandon et al., 2011). One interesting finding of the present study is that ARNI could inhibit the activation of the Wnt/β-catenin pathway, and the expression of sFRP-1 was also increased *in vivo*. We speculated that the inhibition of the Wnt/β-catenin pathway by ARNI might be achieved through sFRP-1. To further verify our hypothesis, we stimulated myocardial fibroblasts with AngII *in vitro*, after pre-incubation with lcz696 and Val. Interestingly, the expression of β-catenin was the lowest when 30 μmol/L of ARNI was administered. Additionally, the inhibition of sFRP-1 led to an increased expression of β-catenin. These observations provide novel insights into the mechanism by which inhibition of the Wnt/β-catenin signaling pathway by ARNI might be achieved by influencing the expression of sFRP-1.

Although our study provides new insights into the mechanism of action of ARNI in myocardial fibrosis, the underlying molecular mechanisms are not well understood. Further investigation is required to explore the specific mechanism of the regulation of the Wnt/β-catenin signaling pathway by ARNI.

## Conclusion

This study confirmed that the Wnt/β-catenin signaling pathway is activated in the context of MI. Importantly, we show that ARNI improves myocardial fibrosis, prevents myocardial remodeling, and inhibits the Wnt/β-catenin signaling pathway via the upregulation of sFRP-1 in the context of MI.

## Data Availability

The datasets presented in this study can be found in online repositories. The names of the repository/repositories and accession number(s) can be found in the article/Supplementary Material.

## Abbreviations

A, late peak; AngII, angiotensin II; ARNI, the angiotensin receptor-neprilysin inhibitor sacubitril/valsartan; CFS, cardiac fibroblasts; DBP, diastolic blood pressure; E, early peak; ELISA, enzyme-linked immunosorbent assay; FS, fractional shortening; H&E, hematoxylin and eosin; HR, heart rate; LCA, left coronary artery; LVEF, left ventricular ejection fraction, LVEDd, left ventricular end-diastolic dimension; LV mass, left ventricular mass; MI, myocardial infarction; SBP, systolic blood pressure; sFRP-1, secreted frizzled-related protein 1;
